# The Vicious Circle of Metabolic Dysfunction-Associated Steatotic Liver Disease When Micronutrient Deficiency Drives Microbial Imbalance and Liver Injury

**DOI:** 10.3390/life15111764

**Published:** 2025-11-18

**Authors:** Iulia Cristina Marginean, Sergiu Marian Cazacu, Mihaela Popescu, George Alexandru Iacob, Larisa Daniela Sandulescu, Sevastita Iordache, Cristina Maria Marginean, Cristin Constantin Vere

**Affiliations:** 1 Doctoral School, University of Medicine and Pharmacy of Craiova, 200349 Craiova, Romania; iulia.cristina18@yahoo.com; 2 Research Center of Gastroenterology and Hepatology, University of Medicine and Pharmacy of Craiova, 200349 Craiova, Romania; sevastita@gmail.com (S.I.); vere_cristin@yahoo.com (C.C.V.); 3 Department of Endocrionology, University of Medicine and Pharmacy of Craiova, 200349 Craiova, Romania; mihaela.n.popescu99@gmail.com; 4 Department of Radiology and Medical Imaging, Clinical Emergency County Hospital Craiova, 200642 Craiova, Romania; georgeicb5@gmail.com; 5 Department of Internal Medicine, University of Medicine and Pharmacy of Craiova, 200349 Craiova, Romania; larisasandulescu@yahoo.com

**Keywords:** MASLD, micronutrient deficiency, gut microbiota, antioxidants, inflammation

## Abstract

Metabolic dysfunction-associated steatotic liver disease (MASLD) is an escalating global health burden and a leading cause of chronic liver disease. Without intervention, MASLD can progress to steatohepatitis, fibrosis, cirrhosis, and hepatocellular carcinoma. Although lifestyle modification is the cornerstone of management, specific dietary patterns are a primary driver of its development. The progression of MASLD is closely linked to micronutrient status, as these nutrients are critical for key biological functions such as antioxidant defense and immune regulation. Micronutrient deficiencies—particularly in essential vitamins and minerals—have been widely studied as independent contributors to MASLD pathogenesis. Similarly, the role of the gut microbiota in disease development has gained attention. However, the interplay between micronutrient deficiencies and gut dysbiosis is often underestimated. Emerging evidence suggests that micronutrient depletion not only directly exacerbates MASLD but also alters gut microbial composition, perpetuating a cycle of metabolic and hepatic dysfunction. This review aims to highlight the bidirectional relationship between micronutrient deficiency and gut microbiome imbalance in MASLD. It explores how dysbiosis impairs the bioavailability of micronutrients, thereby reinforcing a vicious cycle of disease progression. Therefore, effective MASLD management should address both nutritional deficiencies and microbial imbalances. Interventions such as prebiotic and probiotic supplementation may help restore microbial equilibrium and improve micronutrient absorption. Looking forward, personalized therapeutic strategies that combine targeted microbiota modulation with micronutrient repletion may offer promising approaches to curb the rising global burden of MASLD.

## 1. Introduction

As a hepatic component of metabolic syndrome, metabolic dysfunction-associated steatotic liver disease (MASLD) is intimately linked to its hallmark features, including insulin resistance, overweight, and obesity. Patients with MASLD exhibit disturbances in central satiety signaling, resulting in increased intake of macronutrients and subsequent obesity [1]. Additionally, these individuals may experience an energy imbalance partly caused by genetic polymorphisms in metabolic regulatory genes [2,3].

Disruptions in energy balance ultimately cause lipid accumulation in hepatocytes, increasing the risk of developing MASLD (formerly known as nonalcoholic fatty liver disease, NAFLD). In a subset of patients, this condition can progress to metabolic dysfunction-associated steatohepatitis (MASH), fibrosis, and ultimately cirrhosis. It is estimated that the prevalence of both MASLD and obesity affects at least 40% of adults in the United States [4].

Although MASLD can occur in non-obese individuals [5] and some with obesity appear resistant to hepatic steatosis [6], the condition is pathogenically intertwined with obesity. In most affected patients, excessive macronutrient intake drives a state of positive energy balance, characterized by reduced energy expenditure and dysregulated glucose and lipid metabolism. Specifically, dietary components like sucrose and fructose can directly compromise the intestinal epithelium [7]. This breach facilitates the translocation of bacterial products, such as lipopolysaccharides, into the portal circulation. Upon reaching the liver, these molecules activate Toll-like receptor 4 (TLR4), triggering a pro-inflammatory cascade mediated by tumor necrosis factor and promoting liver injury [8,9]. In concert with the development of insulin resistance and dysregulation of adipokines (including adiponectin and leptin), these gut-derived signals are considered essential drivers of MASLD pathogenesis.

This review will critically evaluate the emerging evidence for a self-perpetuating vicious cycle, in which micronutrient deficiency and gut microbiota dysbiosis synergistically drive MASLD progression, and explore the therapeutic implications of disrupting this cycle.

## 2. Material and Methods

This narrative review was conducted to synthesize and critically evaluate the current evidence on the interplay between micronutrient deficiencies, metabolic dysfunction-associated steatotic liver disease (MASLD), and the gut microbiota. A structured search strategy was employed for the identification, selection, and critical appraisal of relevant evidence.

A comprehensive literature search was performed using the PubMed electronic database to identify pertinent English-language articles published up to current date. The search strategy was designed to encompass the three core concepts of this review: micronutrients (specifically vitamin A, D, E, copper, iron, selenium, and zinc), MASLD (and its predecessor term NAFLD, to ensure historical coverage), and the gut microbiota.

The reference lists of key retrieved articles were also manually screened to identify additional relevant publications that may not have been captured in the initial database search.

The initial search results were screened by title and abstract to identify studies relevant to the review’s objective. Full-text articles of potentially eligible studies were then assessed for final inclusion. Given the narrative and integrative nature of this review, the inclusion criteria were designed to capture a broad range of evidence to construct a comprehensive theoretical framework.

Eligible study designs included original research articles (both human observational and interventional studies, and animal model studies), systematic reviews, and meta-analyses. Articles were included if they investigated the relationship between at least two of the three core components (e.g., micronutrient deficiency and MASLD; micronutrient deficiency and gut microbiota; gut microbiota and MASLD), with a strong emphasis on studies that explored the tripartite relationship. Exclusion criteria were: non-English articles; studies not directly relevant to the research question; articles focusing solely on pediatric populations unless explicitly relevant to adult MASLD; studies with insufficient data or methodological quality for assessment; articles published before the year 2000 to focus on contemporary research duplicate publications or duplicate publications.

All identified references were managed using Zotero citation manager (Version 7.0.24) to facilitate the organization of literature, de-duplication of records, and generation of the bibliography in the required citation style.

To visually summarize the complex pathways and interrelationships discussed in this review, schematic illustrations and flowcharts were created. The figures were designed using Adobe Illustrator (Version 27.5.0.695) and Draw.io to ensure clarity and professional presentation.

## 3. Synergistic Mechanisms Between MASLD, Micronutrients, and Microbiota

**Figure 1:**
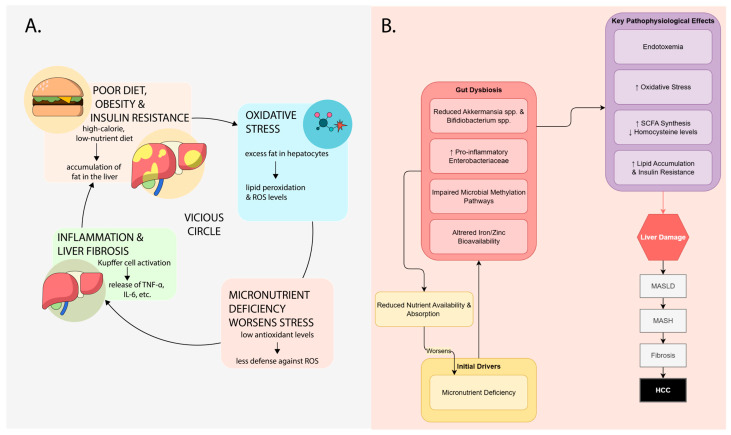
Integrated model of micronutrient deficiency in MASLD. (**A**) Conceptual framework of the self-reinforcing cycle between nutrient status and liver disease. (**B**) Detailed mechanisms of action for individual micronutrients within this framework. SCFA, short-chain fatty acid. MASLD, metabolic dysfunction-associated steatotic liver disease. MASH, metabolic-associated steatohepatitis. HCC, hepatocellular carcinoma.

This review synthesizes evidence supporting a unifying model for MASLD progression: a self-perpetuating cycle between micronutrient deficiency and gut microbiota dysbiosis. In this framework, illustrated in Figure 1, nutrient deficiencies promote gut dysbiosis and barrier dysfunction, driving endotoxemia and hepatic inflammation. This liver injury subsequently worsens metabolic function and nutrient handling, reinforcing the initial deficiencies and accelerating disease progression from steatosis to steatohepatitis and fibrosis.

Key mechanisms that characterize MASLD, including oxidative stress, alterations in glucose and lipid metabolism, glucotoxicity, lipotoxicity, and inflammation, are significantly influenced by both macro- and micronutrients. Growing evidence links nutrient intake to MASLD, showing that diet influences glucose, lipid, and protein metabolism as well as antioxidant defenses, immune modulation, and processes related to carcinogenesis. Consequently, lifestyle changes—such as caloric restriction, low-carbohydrate or low-fat diets, and increased physical activity to promote weight loss—are among the most effective strategies for managing MASLD [10].

The role of micronutrients in MASLD pathogenesis remains complex, as dietary intake is difficult to disentangle from physiological processes that regulate their serum levels. A critical, yet unresolved, question is how hepatic micronutrient dynamics—within both intracellular and extracellular compartments—influence the progression from simple steatosis to steatohepatitis and fibrosis.

Noninvasive biomarkers being evaluated for MASLD staging have so far produced inconsistent and inconclusive results [11,12]. For instance, ferritin and ceruloplasmin can help predict MASLD severity [13,14], while deficiencies in vitamins A and D are associated with more advanced disease [15]. A multicenter study by Neuschwander-Tetri et al. found that circulating levels of these and other fat-soluble vitamins may predict response to novel MASLD therapies that target bile-acid signaling, such as FXR ligands [16,17]. Overall, multiple studies report substantial variability in both micronutrient and macronutrient status among patients with MASLD.

Patients with MASLD often consume diets low in micronutrients, making them susceptible to vitamin and mineral deficiencies [18]. Furthermore, alterations in the gut microbiome can significantly impact micronutrient absorption, which in turn contributes to preexisting vitamin deficiencies. For example, in obese patients with MASLD, inflammatory adipokines released from dysfunctional adipose tissue may further exacerbate these deficiencies. These complex interactions underscore the need to address vitamin deficiencies in the management and improvement of health outcomes for patients with MASLD [19].

The role of micronutrient metabolism—particularly involving vitamins A, D, and E, and minerals such as iron, copper, selenium, and zinc—in the pathogenesis of MASLD underscores its nature as a systemic disorder affecting energy, glucose, and lipid homeostasis. Emerging evidence highlights how micronutrient deficiencies can disrupt the gut microbiome, while dysbiosis, in turn, impairs micronutrient absorption, creating a self-perpetuating cycle that exacerbates MASLD. Understanding these interactions forms the foundation of a holistic approach to this increasingly prevalent condition, offering valuable insights into its underlying mechanisms and opening new avenues for therapeutic strategies, both in adult and pediatric populations worldwide. The interconnection between MASLD, micronutrient deficiency, and microbiota represents an often-underestimated connection with major implications for health.

**Figure 2:**
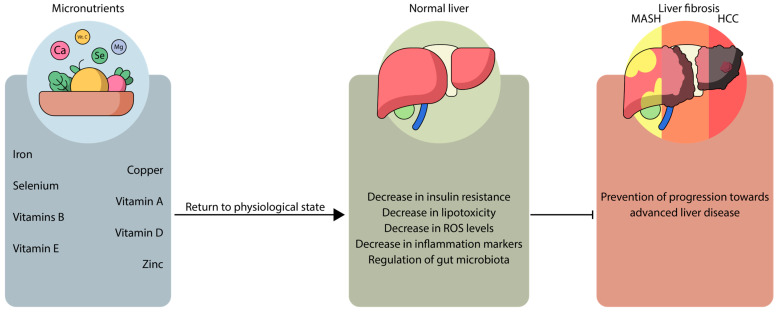
Protective role of micronutrients against liver disease. ROS, reactive oxygen species. MASH, metabolic-associated steatohepatitis. HCC, hepatocellular carcinoma.

Recent studies have underscored the critical role of certain micronutrients in significant immunoinflammatory and metabolic processes associated with the development and management of MASH. These micronutrients have been thoroughly researched and are currently the subject of various clinical trials, given their promising potential in treating MASLD and preventing further progression towards advanced liver disease as illustrated in Figure 2 [20,21].

The gut microbiome functions as a crucial ecosystem that significantly influences human health and disease. Any disruption in its balance—resulting from genetic variations, nutritional intake, lifestyle choices, and other factors—can lead to dysbiosis, which is associated with chronic diseases. Increasing the consumption of micronutrients and other bioactive compounds found in food can help reshape the gut microbiome and may thus play a protective role in preventing chronic diseases.

## 4. Deficiency of Fat-Soluble Vitamins A, D, and E and Their Relationship with MASLD and Microbiota

Rautiainen et al., in a global review, demonstrated that vitamin deficiency is a global health problem with widespread impact [22]. In obesity-related metabolic diseases, most vitamins are deficient, and in MASLD, the main affected vitamins are the fat-soluble ones: A, D, and E, according to the study by Thomas-Valdés et al. Patients with MASLD frequently have diets that are nutrient-poor and high in meat, protein, fat, and sodium [19]. As a result, they are susceptible to lower levels of essential vitamins unless they receive adequate supplementation. Furthermore, deficiencies in these vitamins contribute to low-grade inflammation, which is worsened by the release of inflammatory adipokines from adipose tissue, thereby advancing the progression of metabolic liver disease. This intricate relationship highlights the critical need to address vitamin deficiencies in order to manage and enhance health outcomes for individuals with MASLD.

### 4.1. Vitamin A

Clinical evidence firmly positions vitamin A deficiency within the vicious cycle of MASLD, demonstrating both a correlation with disease severity—such as reduced retinoic acid levels and increased fibrosis-related mortality [23,24]—and a suite of distinct, direct hepatoprotective mechanisms. These include the modulation of lipid metabolism [25,26], antioxidant [27,28] and anti-inflammatory effects [29], and the enhancement of insulin sensitivity [30]. Normally, this fat-soluble vitamin is stored in hepatic stellate cells [31], but its systemic and hepatic levels are intricately linked to its complex absorption process in the gut.

The absorption of dietary vitamin A (as carotenoids and retinoids) is a critical, multi-step process occurring in the upper small intestine [32]. Retinyl esters must be hydrolyzed to retinol by the pancreatic enzymes or mucosal retinyl ester hydrolase [33]. Subsequently, the solubilization of retinol into micelles—a process crucially dependent on bile salts [34]—is a prerequisite for absorption. While carotenoids are absorbed via passive diffusion, retinoids require specific transporter proteins [32].

This process is vital for maintaining intestinal barrier integrity. Vitamin A is essential for regulating the proliferation and differentiation of immune cells in the intestinal epithelium, thereby enhancing resistance to pathogen invasion and preserving the physical barrier formed by epithelial cells and commensal bacteria [35,36]. A compromised barrier is linked to inflammatory diseases and inadequate nutrient absorption [37].

The gut microbiota plays a dual role in influencing vitamin A and carotenoid status. Certain bacteria, including genera like *Lactobacillus* and *Bifidobacterium*, produce secondary bile salts that can enhance the micellarization and absorption of vitamin A [37,38]. More directly, some gut bacteria possess the metabolic machinery to synthesize carotenoids and retinoids de novo from Acetyl-CoA via the mevalonate pathway [39,40]. Furthermore, bacteria such as *Bacteroides* and *Enterococcus* carry *brp/blh* genes that enable the conversion of β-carotene to all-trans retinal [41], a key precursor to retinoic acid.

Conversely, vitamin A can reciprocally shape the gut microbial community. Studies in mouse models have shown that vitamin A status can influence the abundance of immunomodulatory bacteria like segmented filamentous bacteria, which are known to promote the differentiation of Th17 cells [42,43,44]. This highlights a bidirectional relationship where the gut microbiota influences vitamin A bioavailability, and vitamin A, in turn, modulates the gut immune environment.

The evidence linking vitamin A deficiency to MASLD severity is compelling at the clinical correlation level [23,24], but the mechanistic chain of causality remains less defined. The hepatoprotective effects of vitamin A—including modulation of lipid metabolism [25,26] and anti-inflammatory properties [29]—are primarily derived from pre-clinical animal models. While these studies are essential for hypothesis generation, they do not yet establish efficacy in human disease. A significant gap is the lack of interventional trials testing whether vitamin A supplementation can directly improve histologic outcomes in MASLD. Furthermore, the intriguing bidirectional relationship with the gut microbiota is almost entirely based on animal and in vitro evidence [42,43,44]. The critical question of whether vitamin A repletion can therapeutically reshape the human gut microbiome to break the “vicious cycle” in MASLD is completely unexplored. Future research must prioritize human trials that move beyond correlation to establish causation and define the therapeutic potential of vitamin A within the gut–liver axis.

### 4.2. Vitamin D

Vitamin D deficiency correlates with MASLD severity and mortality, yet its therapeutic potential remains mired in contradictory evidence, highlighting a critical disconnect between association and intervention. The biological plausibility for its role is strong, mediated through the vitamin D receptor (VDR) which regulates hepatic inflammation and fibrogenesis [45,46,47,48,49,50]. This positions the vitamin D-VDR axis as a key modulator in MASLD [51,52], with consistent observational data linking deficiency to advanced steatosis and fibrosis [53,54,55] and higher mortality [51,56], while sufficiency appears protective [57].

The therapeutic promise of these findings remains unfulfilled. The body of evidence, as summarized in Table 1, presents a complex and often contradictory picture. While the pre-clinical and epidemiological data are robust, interventional trials have largely failed to demonstrate consistent therapeutic benefits from vitamin D supplementation. This discrepancy underscores a critical gap in our understanding, highlighting that the relationship between vitamin D and MASLD is likely modulatory rather than solely causative, and may be influenced by factors such as VDR polymorphism, baseline deficiency status, and parallel lifestyle interventions. Inconsistent outcomes across vitamin D intervention trials likely stem from substantial heterogeneity in study design and participant characteristics. Variations in baseline vitamin D status, supplementation dose, frequency, and duration, as well as differences in population demographics, comorbidities, and genetic factors influencing vitamin D metabolism, contribute to divergent findings. Moreover, inconsistent outcome measures, inadequate control of confounding factors such as sunlight exposure, dietary intake, and physical activity, along with variable adherence and short follow-up durations, further complicate interpretation. Collectively, these factors highlight the need for rigorously designed, standardized trials that stratify participants by baseline vitamin D status and incorporate comprehensive control of confounding variables to accurately elucidate the therapeutic efficacy and safety of vitamin D supplementation [58,59].

The collective evidence on vitamin D, as summarized in Table 1, presents a classic example of the hierarchy of evidence and the distinction between association and intervention. The consistent findings from large observational meta-analysis [61,62] and the Mendelian randomization study [60]—which suggest a causal, protective effect—provide a strong rationale for a biological link. However, the failure of randomized controlled trials to demonstrate consistent benefit is a major hurdle [64,65,66]. This contradiction can be interpreted in several ways: it may indicate that low vitamin D is a marker of liver dysfunction rather than a modifiable driver, or that RCTs supplement too late in the disease process. The evidence strongly supports vitamin D as a biomarker for MASLD risk and progression, but the current strength of evidence is insufficient to support its use as a primary therapy. The most promising future direction may be to target vitamin D repletion in deficient, early-stage MASLD patients with specific VDR genotypes, rather than employing blanket supplementation.

#### Gut Microbiota in Vitamin D Interaction with MASLD

The gut microbiota is a key contributor to the onset and progression of MASLD, with vitamin D deficiency representing a critical factor in its dysregulation. This deficiency has been strongly associated with intestinal dysbiosis, which can impair nutrient absorption and alter the enterohepatic circulation of bile acids. Dietary patterns further exacerbate this cycle; a diet rich in fats and carbohydrates elevates circulating lipopolysaccharides (LPS), promoting hepatic insulin resistance [67], immune activation, and systemic inflammation [68]. Furthermore, such dysbiosis increases intestinal permeability, fostering a state of chronic inflammation in individuals with obesity, metabolic syndrome, and MASLD [69].

The relationship between the liver and gut microbiota is bidirectional. Liver damage impairs vitamin D synthesis, which subsequently disrupts the function of Paneth cells in the small intestine. This disruption leads to intestinal dysbiosis and promotes liver fibrogenesis, creating a vicious cycle [70]. Mechanistically, vitamin D deficiency upregulates key pathways related to oxidative stress (e.g., heme oxygenase) and inflammation (e.g., IL-6, IL-1β) via Toll-like receptor signaling. This directly connects impaired vitamin D metabolism to both hepatic lipotoxicity and the liver’s pathogenic response to gut-derived microbial translocation [71].

Given this framework, vitamin D emerges as a potential modulator of the gut–liver axis. In a MASLD rat model, vitamin D was shown to reverse high-fat diet-induced dysbiosis, specifically by increasing *Lactobacillus* and decreasing *Acetatifactor*, *Oscillibacter*, and *Flavonifractor* [72]. This suggests that vitamin D can attenuate MASLD by altering microbial composition. A more recent integrated analysis confirmed that vitamin D treatment suppresses HFD-induced MASLD and corrects gut dysbiosis, positioning supplementation as a potential therapeutic strategy targeting specific gut microbiota populations [73].

The modulatory effects of vitamin D are likely mediated through multiple mechanisms, including the regulation of antimicrobial peptide expression and its protective role in maintaining intestinal mucosal integrity and promoting epithelial healing [74,75,76]. Supporting a targeted interaction, a clinical study by Jones et al. revealed a potential link between vitamin D and *Lactobacillus reuteri*, possibly facilitated through bile acid metabolism, highlighting a specific pathway with direct probiotic implications [77].

### 4.3. Vitamin E

Vitamin E stands apart as the only micronutrient with a formal AASLD recommendation for treating non-cirrhotic MASH, a status supported by RCTs and underpinned by its potent antioxidant and anti-inflammatory properties [78,79,80]. This clinical relevance is underscored by the consistent finding of depleted plasma and hepatic vitamin E levels in patients with MASLD and MASH [81,82]. Experimentally, its hepatoprotective profile is multifaceted, demonstrating antisteatotic effects—such as inhibiting the fatty acid transporter CD36 to reduce lipid uptake [78]—as well as anti-inflammatory and antifibrotic actions, the latter mediated by downregulating TGF-β1 and procollagen genes [83]. This combination of hepatoprotective properties has formed the foundation for its therapeutic application in MASH [84,85].

Vitamin E deficiency commonly arises from fat malabsorption, particularly in children with cholestatic liver disease, pancreatitis, or cystic fibrosis, due to impaired secretion of bile and pancreatic enzymes essential for lipid digestion. In adults, it may also result from intestinal dysbiosis [86]. The impact of the jejunal and colonic microbiota on vitamin E metabolism remains largely underexplored. Ran et al. (2019) investigated the role of gut microbiota in vitamin E bioavailability and found that antibiotic treatment in a mouse model increased vitamin E absorption, suggesting that certain intestinal commensals may degrade the vitamin [87]. Additionally, another study showed that dietary intake of vitamin E has been positively correlated with short-chain fatty acid (SCFA) production and the relative abundance of beneficial microbial taxa, including *Akkermansia*, *Lactobacillus*, *Bifidobacterium*, and *Faecalibacterium* [88].

Vitamin E stands out as the only micronutrient with a formal recommendation (AASLD) for treating non-cirrhotic MASH, a status supported by RCTs [84,85]. This places the evidence for vitamin E’s efficacy in a specific patient population on a much stronger footing than for other micronutrients. However, this strength is tempered by important caveats. The benefits are not universal, and the mechanistic evidence—such as the inhibition of fatty acid transporter CD36—is largely derived from animal models [78]. Furthermore, the relationship between vitamin E and the gut microbiota is particularly underdeveloped. The finding that antibiotic treatment increases vitamin E absorption in mice suggests a complex and potentially adversarial relationship with certain gut bacteria, which contrasts with the positive correlations seen with SCFA producers [87,88]. A significant gap is the lack of human studies exploring whether the therapeutic benefit of vitamin E in MASH is partially mediated through measurable changes in the gut microbiota. Understanding this interaction could help identify responders and optimize combination therapies.

## 5. Mineral Deficiency in MASLD and the Interrelationship with Microbiota

### 5.1. Copper

Copper plays a complex and underappreciated role in MASLD, where the implications of both deficiency and excess contribute to hepatic fibrosis. Its absorption in the intestine, beginning with the reduction in dietary Cu^2+^ by cytochrome B reductase [89,90,91], is a tightly regulated process mediated by copper transporter 1 (CTR1). Once in hepatocytes, copper is incorporated into ceruloplasmin and distributed systemically before biliary excretion [89,92]. This micronutrient is essential for fundamental processes including cellular energy homeostasis, ROS detoxification, and immunometabolic regulation [93,94,95,96,97]. Copper deficiency has been linked to an increased risk of MASLD, MASH, and liver fibrosis through disrupted energy homeostasis, impaired lipid metabolism, a pro-inflammatory prostaglandin profile, enhanced lipid peroxidation, and reduced antioxidant defenses.

Morrell et al. highlighted in their study that copper deficiency is a risk factor for conditions characterized by mitochondrial dysfunction and altered lipid metabolism, including MASLD [98]. Oxidative stress is also a critical factor in the complex pathogenesis of MASLD. From this perspective, Ma Y. demonstrated the importance of antioxidants in managing the disease [99]. Notably, superoxide dismutase (SOD), a key antioxidant enzyme, requires sufficient copper for its activity [94], suggesting that reduced copper availability may impair antioxidant defenses and thereby increase the risk of MASLD and its associated vascular complications.

Studies in copper-deficient mouse models reveal that a lack of copper, even in the absence of obesity, exacerbates MASLD progression. For instance, research by Song et al. demonstrated that copper-deficient mice fed a high-fructose diet exhibited increased lipogenesis and developed biochemical, gene expression, and histological hallmarks of MASLD and MASH [93,100]. Furthermore, reduced copper bioavailability contributes to pathological iron retention in the liver [101]. These findings collectively support the exploration of dietary copper supplementation as a potential therapeutic strategy for MASLD patients with confirmed copper deficiency. A growing body of evidence underscores the crucial role of copper homeostasis in the pathogenesis of hepatic steatosis [102,103,104].

Several studies have indicated that copper may play a role in regulating gut microbiota and maintaining gut barrier function [105]. Evidence suggests that certain bacterial genera influence copper homeostasis, including its absorption, storage, regulation, and excretion. For instance, *Akkermansia* appears to assist the host in managing copper levels, potentially through interactions with the gut barrier, modulation of the microbiota, and effects on copper chelation, metabolism, and absorption [106]. In rats given different dietary copper/fructose combinations, *Lactobacillus* abundances increased under copper supplementation [105].

Song et al. demonstrated in a study that dietary alterations in copper levels lead to a decrease in *Bifidobacterium* abundance. Although this change does not appear to enhance copper absorption, it reflects the impact of copper on the gut microbiome [105].

Studies on copper supplementation have reported increases in certain microbial taxa. For example, in rat studies, copper bound to *Bacillus subtilis* enhanced offspring survival and promoted the development of duodenal villi. These beneficial effects were associated with increased abundances of *Lachnospiraceae*, *Ruminococcaceae*, and *Intestinibacter* [105]. Further studies are needed to emphasize the connection between microbiota and copper, and their involvement in MASLD progression.

The role of copper in MASLD is paradoxical and under-investigated. The evidence for copper deficiency exacerbating steatosis and fibrosis is robust in animal models [93,100], and the biochemical rationale—its essential role in antioxidant defense via SOD [94]—is sound. However, the translational leap to human MASLD is fraught with complexity. Human data are primarily associative, and it remains unclear whether copper deficiency is a common cause or a consequence of advanced liver disease. The interaction with the gut microbiota is a novel concept, but current evidence is preliminary, based on rodent studies showing microbial shifts in response to dietary copper [105]. A critical, unanswered question is whether copper status is a measurable and modifiable risk factor in human MASLD. Well-designed human studies are needed to determine the prevalence of deficiency in patients and to test if targeted supplementation in a deficient subpopulation can alter disease course, without risking additional toxicity [104].

### 5.2. Iron

Iron dysregulation, particularly systemic overload, is increasingly recognized as a key contributor to MASLD pathogenesis. Recent studies, including Mendelian randomization, suggest a causal link, with excessive dietary iron exacerbating inflammation and disrupting lipid metabolism [107,108,109]. This is often indicated by hyperferritinemia, which correlates with more severe metabolic dysfunction and liver injury [110]. The dysregulation is driven by multiple factors: genetic predispositions like abnormal hepcidin release can increase intestinal absorption [111], while the MASLD metabolic milieu itself, with excess free fatty acids, promotes hepatic iron uptake via IRP1 and TfR-1 pathways [112]. A critical regulator in the hypoxic gut environment is hypoxia-inducible factor 2 alpha (HIF-2α), which upregulates iron absorption proteins like divalent metal transporter 1 (DMT1) [113]. The resulting iron overload drives detrimental processes such as ferroptosis—an iron-dependent cell death—which triggers inflammation in steatohepatitis and causes oxidative DNA damage [114].

This dysfunctional iron metabolism interacts synergistically with dietary insults. A recent 2023 study by Zhang et al. demonstrated that a high-fat diet exacerbates lipid metabolism disorders and liver injury, a process driven by iron overload and oxidative stress [115]. The mechanism of iron-induced damage is further illuminated by Gao H. et al., who found that hepatocytes under lipotoxic stress release iron-containing extracellular vesicles. This process paradoxically creates hepatic iron deficiency while promoting iron accumulation in hepatic stellate cells, thereby directly fueling hepatic steatosis and fibrosis [116].

Recent comprehensive studies by Seyoum and Mayneris-Perxachs have shown that the complex interplay between gut microbiota and the host not only affects the progression of MASLD but also impacts iron homeostasis [117,118]. This interaction creates a detrimental cycle where iron overload promotes lipid accumulation via mitochondrial dysfunction triggered by oxidative stress [119].

Iron absorption is tightly regulated in the duodenum [120], with heme and non-heme iron absorbed through distinct mechanisms [121,122]. Heme iron is taken up by heme carrier protein 1 located on the duodenal brush border membrane, while non-heme iron, which is not readily bioavailable, must first be reduced to its ferrous form by duodenal cytochrome b before being transported by the divalent metal transporter 1 across the brush border. Additionally, iron absorption also occurs in the ileum and colon [123]. Hepcidin expression increases during systemic inflammation, leading to reduced iron bioavailability [124]. Pathogenic bacteria can trigger such inflammation, further limiting intestinal iron availability. These bacteria obtain iron through various strategies, including the secretion of siderophores—specialized ferric chelators—absorption of ferrous iron after ferric iron reduction, or utilization of host iron-binding proteins like transferrin. The ferric-siderophore complexes are then taken up by specific bacterial membrane proteins [125].

Bacteria can also exploit siderophores produced by other microbes by expressing outer membrane receptors with diverse ligand-binding sites. Siderophore production is triggered by bacterial iron deficiency and typically does not occur in iron-replete environments [126]. It is important to note that much of our understanding of bacterial iron uptake derives from studies on pathogenic species. Traditionally, certain beneficial bacteria, such as those from the genus *Lactobacillus*, were thought not to require iron for growth. However, specific iron acquisition systems have been identified in *Lactobacillus plantarum* and *Lactobacillus sakei* [127,128]. Given that *Lactobacillus* species are abundant in the small intestine—where iron absorption is most active—further research is warranted to clarify their potential role in influencing host iron bioavailability.

The gut microbiota significantly influences host iron bioavailability through multiple mechanisms. Certain commensal bacteria produce promoters of iron solubility, such as lactic acid, throughout the digestive tract. Furthermore, bacterial fermentation of indigestible carbohydrates generates short-chain fatty acids (SCFAs), which lower the luminal pH. This acidic environment, as highlighted by Biesalski HK, can degrade micronutrient-chelating complexes and facilitates the reduction of ferric iron (Fe^3+^) to the more soluble ferrous form (Fe^2+^), thereby enhancing iron absorption for both bacteria and the host [129,130].

This relationship is not unidirectional; the host actively regulates bacterial iron access. During inflammation, for instance, the host produces lipocalin-2, which binds to bacterial siderophores to sequester iron and limit bacterial growth [131]. This dynamic underscores a complex interplay, moving beyond a simple competition model. Recent research suggests that commensal bacteria have evolved sophisticated strategies not only to acquire and release iron but also to facilitate its sharing with other microbes and the host, indicating a more cooperative dimension to iron homeostasis within the gut ecosystem [131,132].

The role of iron in MASLD is contradictory; while the literature focuses on iron overload as a driver of disease progression, mechanistic studies often reveal a complex dysregulation rather than simple excess. The most compelling evidence for a causal role comes from a Mendelian randomization study, suggesting a causal link between iron status and MASLD [108]. This is strongly supported by interventional animal models where dietary iron overload directly exacerbates liver injury and inflammation [109,115]. However, in humans, the data are primarily observational, correlating hyperferritinemia with disease severity without proving direct causation [110]. The interaction with the gut microbiota adds a significant layer of complexity. While the mechanisms of bacterial iron acquisition are well-studied in pathogens, their role in the commensal gut ecosystem and the subsequent impact on host iron bioavailability in MASLD remain speculative, based largely on in vitro and pre-clinical inferences [125,126,127,128]. A critical unanswered question is whether therapeutic modulation of iron levels can consistently improve MASLD outcomes and, if so, whether this effect is mediated by altering the gut microbial community.

### 5.3. Selenium

Selenium, an essential micronutrient, performs a variety of vital functions in the human body, including antioxidant defense, cancer prevention, and immunomodulation [133]. These roles, linked to its structural and enzymatic functions, have been extensively documented in studies such as the one by Hariharan et al. [134]. Additionally, Gupta et al. emphasize selenium’s important implications in metabolic diseases [135]. A six-year study by Wang et al. further revealed the complex relationship between selenium and MASLD, demonstrating non-linear associations between serum selenium levels, ALT activity, and MASLD prevalence [136].

A recent study by Liu et al., published in 2022, found that lower blood selenium levels are linked to an increased risk of advanced liver fibrosis [137]. Conversely, another study reported a positive correlation between higher blood selenium levels (above approximately 130 μg/L) and MASLD, suggesting a dose–response relationship [138]. In experimental models, selenium supplementation in MASLD mice showed beneficial effects, including reduced liver injury, decreased oxidative stress, improved insulin sensitivity, and lower inflammation [139,140]. While selenium is a cofactor of several antioxidant enzymes against cancer and is essential for human health, its excess intake may also be harmful. A recent study showed a U-shaped association between selenium intake and cancer risk, and data from randomized clinical trials did not support the protective effect of selenium administration against some cancers, including colorectal, non-melanoma, skin, lung, breast, bladder, and prostate cancer [141]. Thus, it is critical to note that the range between an essential level and a toxic level of selenium is narrow, and the range of safe intake for selenium is still not well-defined [142]. Further mechanistic investigation is warranted to understand better a U-shaped association between selenium intake and cancer risk.

Selenium availability significantly impacts gut microbial composition and function [133]. Certain bacteria, such as *Clostridium* and *Bacteroides*, incorporate selenium into bacterial selenoproteins. Selenium deficiency can reduce microbial diversity, promote the growth of pro-inflammatory bacteria, and decrease populations of beneficial SCFA-producing microbes. Conversely, selenium supplementation may help restore microbial balance by increasing bacterial diversity and boosting levels of SCFA producers, including species like *Lactobacillus* and *Bifidobacterium*. However, excessive selenium intake might have adverse effects, potentially disrupting this balance [133,143].

The relationship between selenium and MASLD is a prime example of the non-linear, “U-shaped” association, where both deficiency and excess appear harmful [137,138]. The evidence for this is primarily cross-sectional and observational, which cannot establish causality. It is plausible that selenium status is a marker of overall dietary quality or liver function rather than a direct driver of disease. The beneficial effects of supplementation seen in animal models have not been consistently demonstrated in humans and are counterbalanced by data linking high levels to increased MASLD prevalence [139,140]. This creates a significant clinical dilemma. The strength of evidence is currently insufficient to recommend routine selenium testing or supplementation in MASLD patients. The primary gap in knowledge is a mechanistic understanding of why excess selenium is harmful in the context of hepatic steatosis and the lack of long-term, prospective studies or Mendelian randomization analyses to clarify the direction of causality.

### 5.4. Zinc

Zinc plays essential roles in numerous biological processes, including antioxidant defense, immune regulation, insulin signaling, and maintaining intestinal barrier integrity. Zinc deficiency leads to increased oxidative stress, inflammation, and insulin resistance, all of which contribute to the progression of MASLD. Zinc influences MASLD both directly—by affecting liver metabolism—and indirectly—through modulation of the gut microbiota and strengthening of the intestinal barrier. It promotes the expression of tight junction proteins and mucins, activates metallothioneins and SOD, inhibits NF-κB and other pro-inflammatory cytokines, enhances beta-oxidation, and suppresses de novo lipogenesis [144].

Zinc absorption takes place primarily in the duodenum, after which it is transported to sites of metalloprotein synthesis via zinc-regulated transporters ZnT2-10 or exported into the bloodstream by ZnT1 [145]. Unlike iron, excess zinc can be actively excreted. Unabsorbed zinc reaching the colon may be absorbed by colonocytes, which also express zinc transporters [146].

Zinc deficiency is a well-established clinical factor in chronic diarrhea [146], a link that has prompted investigation into its effects on the gut microbiota, primarily in animal models [147]. Zinc supplementation exhibits antimicrobial properties that alter bacterial populations throughout the gastrointestinal tract [148]. These alterations are complex and context-dependent; for instance, studies report conflicting shifts in Enterobacteriaceae and lactic acid bacteria, showing either a reduction or an increase [149]. Notably, one study identified the genus *Ruminococcus* as a potential biomarker for predicting the host’s zinc requirements [148]. Furthermore, these zinc-induced compositional changes have functional metabolic consequences, as evidenced by increased colonic levels of SCFAs [144].

Zinc status plays a critical role in shaping gut microbiota composition and function. Zinc deficiency reduces microbial diversity, decreasing beneficial taxa such as *Lactobacillus* and *Bifidobacterium*, while promoting dysbiosis favoring inflammation, with an increase in *Enterobacteriaceae*, *Clostridium*, and other potential pathogen species [150,151,152]. This imbalance is associated with increased intestinal permeability, favoring endotoxin translocation with consequently liver damage and inflammation. Recent studies showed that an adequate zinc intake supports the growth of commensal and SCFA-producing bacteria (*Akkermansia*, *Roseburia*) [152].

Zinc deficiency is a compelling, multi-systemic mechanism for exacerbating MASLD, impacting insulin signaling, oxidative stress, and intestinal barrier integrity [144]. However, the evidence is heavily skewed towards pre-clinical and animal studies. While the associations in human MASLD are plausible, they are not yet proven to be causal. The most promising aspect of zinc is its direct and modifiable interaction with the gut microbiota. Studies show that zinc supplementation can selectively promote beneficial bacteria and increase SCFA production, providing a tangible pathway to break the “vicious cycle” [144,152]. Zinc represents a high-potential, but currently unproven, therapeutic target within the gut-liver axis.

## 6. Clinical Implications and Future Perspectives

The intricate vicious cycle between micronutrient deficiency, gut dysbiosis, and MASLD progression, as detailed in this review, underscores the limitations of a one-size-fits-all therapeutic approach. While lifestyle modification remains the undisputed cornerstone of MASLD management, a deeper understanding of these interrelationships opens the door to more nuanced, adjunctive nutritional strategies. Translating this mechanistic knowledge into clinical practice, however, requires careful consideration of current evidence, guideline recommendations, and the design of future trials.

### 6.1. Micronutrient Supplementation: A Double-Edged Sword

The therapeutic potential of micronutrient repletion is not equal across all nutrients and is often fraught with complexity.

Vitamin E stands as a unique case, being the only micronutrient with a formal AASLD recommendation for the treatment of non-cirrhotic, biopsy-proven MASH [84,85]. This endorsement is based on RCTs demonstrating improved histology. However, its clinical application is tempered by concerns regarding long-term administration. Evidence from multiple studies indicates that high-dose Vitamin E supplementation may contribute to adverse health outcomes, including increased all-cause mortality and a higher incidence of hemorrhagic stroke, cardiovascular events, and certain malignancies [153]. Such risks are particularly concerning in individuals with existing cardiovascular or coagulation disorders and emphasize the need for a careful risk–benefit assessment. Therefore, its use must be judicious, targeting a specific patient population, and it cannot be recommended for the general management of simple steatosis.

Vitamin D presents a paradigm of the disconnect between robust observational data and interventional trial outcomes. While Mendelian randomization studies suggest a causal, protective effect of lifelong higher vitamin D status [60], and deficiency is consistently linked to disease severity [61], supplementation trials have largely failed to show consistent benefit [64,65]. Inconsistent outcomes across vitamin D intervention trials likely stem from substantial heterogeneity in study design and participant characteristics. Variations in baseline vitamin D status, supplementation dose, frequency, and duration, as well as differences in population demographics, comorbidities, and genetic factors influencing vitamin D metabolism, contribute to divergent findings. Moreover, inconsistent outcome measures, inadequate control of confounding factors such as sunlight exposure, dietary intake, and physical activity, along with variable adherence and short follow-up durations, further complicate interpretation. This collective evidence highlights that vitamin D deficiency may be more a marker of advanced liver dysfunction than a readily modifiable driver in late-stage disease. Future research must focus on well-designed RCTs that stratify participants by baseline vitamin D status and incorporate comprehensive control of confounding variables to accurately elucidate its therapeutic potential [58,59].

Selenium exemplifies a “U-shaped” dose–response relationship, where both deficiency and excess are associated with negative outcomes [137,138]. Its narrow therapeutic window necessitates caution. While selenium is a cofactor of several antioxidant enzymes and is essential for human health, its excess intake may also be harmful. A recent study showed a U-shaped association between selenium intake and cancer risk, and data from randomized clinical trials did not support the protective effect of selenium administration against some cancers, including colorectal, non-melanoma, skin, lung, breast, bladder, and prostate cancer [141]. Thus, it is critical to note that the range between an essential level and a toxic level of selenium is narrow, and the range of safe intake for selenium is still not well-defined [142]. While animal models show promise [139,140], the association of high selenium levels with increased MASLD prevalence in humans [138] precludes any recommendation for supplementation outside of a confirmed deficiency. The clinical priority should be to avoid deficiency without inducing excess, emphasizing dietary sources over high-dose supplements.

For minerals like zinc and copper, the evidence for deficiency exacerbating MASLD is compelling in pre-clinical models [93,100,144], but human interventional data are scarce. The role of iron is particularly complex, with overload being a clear concern [108,109]. The clinical implication is that routine screening for these deficiencies in high-risk MASLD patients may be warranted, but blanket supplementation is not yet supported by evidence. Repletion should be considered on a case-by-case basis upon confirmation of a deficiency.

### 6.2. Strength of Evidence for Probiotic/Prebiotic Therapies

Emerging evidence suggests that probiotic, prebiotic, and synbiotic interventions may confer modest benefits in patients with MASLD, primarily by improving liver enzymes and reducing hepatic steatosis on imaging. However, the overall strength of this evidence is low to moderate, and the clinical significance remains uncertain. The observed benefits are often small, and the field is characterized by considerable heterogeneity in probiotic strains, formulations, and trial design. Most critically, the evidence is based largely on surrogate markers (e.g., ALT) rather than histologic endpoints, and long-term outcomes are unknown. This directly explains why major international guidelines (EASL, AASLD, APASL) do not currently recommend their routine use. The existing data are sufficient to justify further research but are insufficient to support these interventions as standard of care. Future large-scale RCTs with standardized protocols, defined microbial consortia, and hard histological endpoints are essential to determine if modulating the gut microbiota is a viable therapeutic strategy for MASLD [154,155,156].

While recent findings suggest probiotics and prebiotics may modestly improve liver enzymes and steatosis in MASLD, current guidelines, including EASL (2024), AASLD (2023), and APASL (2025), do not recommend their routine use, emphasizing lifestyle modification as the primary management strategy until further high-quality clinical trials clarify their efficacy and safety, the evidence remaining insufficient for formal guideline endorsement.

### 6.3. Toward a Personalized Nutritional Medicine Approach

The failure of blanket supplementation strategies and the complex, individual interplay between diet, genetics, microbiota, and micronutrient status point unequivocally to the need for personalized medicine in MASLD. A promising strategy involves moving beyond simply measuring serum micronutrient levels. Future frameworks should integrate multi-omics data, combining:

- Comprehensive Nutritional Assessment: Detailed dietary intake and micronutrient status profiling.
- Microbiome Genomic Sequencing: To identify patient-specific dysbiotic patterns that predict nutrient malabsorption (e.g., microbes that degrade vitamins or promote/inhibit mineral absorption) [37,39,87,133].
- Host Genetic Markers: Including polymorphisms in genes related to micronutrient metabolism (e.g., VDR) and MASLD risk (e.g., PNPLA3, HSD17B13).

This integrated profile could then guide highly personalized interventions. For example, a patient with vitamin D deficiency, a dysbiotic gut profile low in *Lactobacillus*, and a specific VDR genotype might be targeted with a combination of vitamin D supplementation and a specific probiotic strain known to interact positively with vitamin D metabolism [77]. Similarly, zinc supplementation could be targeted to patients with deficiency and a gut microbial signature indicating impaired barrier function [144,152].

## 7. Limitations

The study primarily relies on associations between micronutrient deficit and MASLD progression, with limited considerations related to uncontrolled variables such as dietary habits, physical activity, medication use, genetic predispositions, or other lifestyle factors could confound the observed relationships between micronutrient depletion and MASLD progression. Given the current reliance on associative findings, future investigations should focus on elucidating causal mechanisms through well-controlled prospective or mechanistic studies. Integrating nutritional assessments with genetic, metabolic, and lifestyle data will provide a more comprehensive understanding of micronutrient involvement in MASLD pathogenesis.

## 8. Conclusions

This review establishes that the progression of MASLD is driven not only by macronutrient excess but also by a critical, self-perpetuating cycle involving micronutrient deficiencies and gut microbial dysbiosis. This triad creates a pathogenic feedback loop: micronutrient depletion impairs antioxidant defenses and promotes inflammation, which exacerbates liver injury and metabolic dysfunction, while simultaneously altering the gut microbiota in ways that further compromise nutrient absorption and increase endotoxemia. The key clinical takeaway is that the assessment of micronutrient status—particularly for vitamins A, D, E, and minerals like zinc, copper, and selenium—should be integrated into the evaluation of MASLD, as deficiencies are common and correlate with disease severity.

However, translating this knowledge into therapy requires a nuanced approach. With the notable exception of vitamin E for specific cases of non-cirrhotic MASH, the evidence does not support blanket micronutrient supplementation. The risks of high-dose vitamin E, the U-shaped response curve of selenium, and the inconsistent results from vitamin D trials underscore that repletion must be targeted and evidence-based. Similarly, while the gut–liver axis presents a compelling therapeutic target, current international guidelines do not yet endorse the routine use of pro- or prebiotics due to a lack of high-quality evidence demonstrating histological benefit. Ultimately, transcending a purely caloric-centric view to embrace the complexities of micronutrient and microbial health is paramount. The future of MASLD management lies in personalized strategies that combine lifestyle modification with nutritional interventions tailored to an individual’s unique micronutrient status, gut microbiome, and genetic profile to effectively break this vicious cycle.

## Figures and Tables

**Table 1:** Summary of Systematic Reviews and Meta-Analyses on Vitamin D and MASLD.

Author(s) and Year	Type of Review	No. of Studies/Participants	Main Findings	Conclusion	Key Limitations/Context	Reference
Fang et al. (2024)	Mendelian Randomization	133 MR Studies	OR 0.85 (95% CI: 0.73–0.99) per 1-SD increase in genetically predicted 25(OH)D. 15% lower odds of MASLD.	Likely causal, protective effect of lifelong higher vitamin D status on MASLD risk.	MR assesses genetic predisposition, suggesting causality, but does not evaluate the effect of supplementation.	[60]
Liu et al. (2020)	Observational Meta-Analysis	15 Studies/20,096 participants	SMD −0.90 ng/mL (95% CI: −1.29 to −0.52) in MASLD patients. OR 0.64 (95% CI: 0.54–0.77) for MASLD with vitamin D deficiency.	Strong association between lower vitamin D levels and the presence of MASLD.	High heterogeneity (I^2^ = 99%); association stronger in Western populations.	[61]
Zhu et al. (2019)	Observational Meta-Analysis	8 Studies (Pediatric)	SMD −0.59 (95% CI: −0.98 to −0.20) in children/adolescents with MASLD.	Significant association between lower vitamin D and pediatric MASLD (medium effect size).	Focuses specifically on pediatric population.	[62]
Jaruvongvanich et al. (2017)	Observational Meta-Analysis	6 Studies/974 participants (Biopsy-proven)	No significant association with histologic severity (NAS ≥ 5 vs. <5). No significant association with fibrosis stage (F3–F4 vs. F0–F2).	No association between vitamin D levels and histologic severity of MASLD.	Focuses on disease severity among those already diagnosed; biopsy-proven cohorts.	[63]
Sharifi & Amani (2019)	Systematic Review of Clinical Trials	6 Trials/330 patients	Inconsistent results: Only 2/6 studies showed improvement in steatosis/liver enzymes. No effect on fibrosis biomarkers. Effectively raised serum 25(OH)D levels.	Insufficient and inconsistent evidence for Vitamin D supplementation benefits on MASLD pathology.	Highlights the gap between raising serum levels and improving clinical outcomes. Small, short-term trials.	[64]
Bjelakovic et al. (2021)	Cochrane Review (RCTs)	27 RCTs/1979 patients (Various CLD)	Evidence on liver-related mortality is far too uncertain to draw conclusions (very wide CI, small sample).	No reliable evidence for or against vitamin D supplementation in chronic liver diseases.	Includes various liver diseases (MASLD, HCV, cirrhosis); highlights very low quality of evidence for critical outcomes.	[65]
Abe et al. (2021)	Systematic Review (Narrative)	17 Articles	Synthesizes conflicting evidence. Notes some studies show biochemical improvement, but not consistent for histology or enzymes. Proposes VDR genotype/site of activation as key to inconsistent results.	Evidence is mixed. The role of Vitamin D is complex and not yet fully understood.	Provides a mechanistic hypothesis for inconsistencies (e.g., VDR in hepatocytes may promote lipid accumulation).	[66]

MASLD, metabolic dysfunction-associated steatotic liver disease; MR, Mendelian Randomization; RCT, randomized controlled trial; CLD, chronic liver disease; OR, odds ratio; CI, confidence interval; SD, standard deviation; SMD, standardized mean difference; 25(OH)D, 25-hydroxyvitamin D; NAS, NAFLD Activity Score; HCV, hepatitis C virus. VDR, vitamin D receptor.

## Data Availability

No new data were created or analyzed in this study.
